# Intestinal parasitosis and shigellosis among diarrheal patients in Gondar teaching hospital, northwest Ethiopia

**DOI:** 10.1186/1756-0500-4-472

**Published:** 2011-10-31

**Authors:** Kahsay Huruy, Afework Kassu, Andargachew Mulu, Netsanet Worku, Teshome Fetene, Simon Gebretsadik, Fantahun Biadglegne, Yeshambel Belyhun, Abebe Muche, Aschalew Gelaw, Belay Anagaw, Sisay Yifru, Yemataw Wondie, Assegedech Bekele, Moges Tiruneh, Dieter Reissig, Feleke Moges

**Affiliations:** 1 Department of Medical Laboratory Technology, College of Medicine and Health Sciences, University of Gondar, Ethiopia; 2 Institute of Virology, Faculty of Medicine, University of Leipzig, Johannisallee 30, 04103, Leipzig, Germany; 3 Department of Microbiology and Parasitology, College of Medicine and Health Sciences, University of Gondar, Ethiopia; 4 School of Public Health, College of Medicine and Health Sciences, University of Gondar, Ethiopia; 5 Adamitulu PPSC, Research and Development Department, Addis Ababa, Ethiopia; 6 Department of Anatomy, College of Medicine and Health Sciences, University of Gondar, Ethiopia; 7 Department of Pediatrics, College of Medicine and Health Sciences, University of Gondar, Ethiopia; 8 Department of Psychology, Faculty of Social Sciences and Humanities University of Gondar, Ethiopia; 9 Department of Anatomy, Faculty of Medicine, University of Leipzig, Germany

**Keywords:** Intestinal parasitosis, Shigellosis, Gondar

## Abstract

**Background:**

Diarrheal diseases are the major causes of morbidity and mortality in developing world. Understanding the etiologic agents of diarrheal diseases and their association with socio-demographic characteristics of patients would help to design better preventive measures. Thus, this study was aimed to determine the prevalence of intestinal parasites and enteropathogenic bacteria in diarrheic patients.

**Methods:**

A cross-sectional study involving 384 consecutive diarrheal patients who visited Gondar teaching hospital, Gondar, Ethiopia from October 2006 to March 2007 was conducted. Stool specimens were collected and examined for intestinal parasites and enteropathogenic bacteria following standard parasitological and microbiological procedures.

*****Results***:**

Intestinal parasites were diagnosed in 36.5% of the patients. The most frequently encountered protozoan parasite was *Entamoeba histolytica/dispar *(7.3%) followed by *Giardia lamblia *(5.0%), C*ryptosporidium parvum *(1.8%) and *Isospora belli *(1.3%). The dominant helminthic parasite identified was *Ascaris lumbricoides *(5.5%) followed by *Strongyloides stercoralis *and *Schistosoma mansoni *(3.1% each), hookworm infection (1.8%), and *Hymenolepis *species (1.3%). Multiple infections of intestinal parasites were also observed in 6.3% of the patients. Among the enteropathogenic bacteria *Shigella *and *Salmonella *species were isolated from 15.6% and 1.6%, respectively, of the patients. *Escherichia coli O57:H7 *was not found in any of the stool samples tested. Eighty eight percent and 83.3% of the *Shigella *and *Salmonella *isolates were resistant to one or more commonly used antibiotics, respectively.

Intestinal parasitosis was higher in patients who live in rural area, in patients who were washing their hands after visiting toilet either irregularly with soap and without soap or not at all, in patients who used well and spring water for household consumption, and in patients who had nausea (*P *< 0.05). Statistically significant associations were also observed between Shigella infections and patients who were using well and spring water for household consumption, and patients who had dysentery and mucoid stool (*P *< 0.05).

**Conclusions:**

The high prevalence of intestinal parasites and *Shigella *species in diarrheic patients calls for institution of appropriate public health intervention measures to reduce morbidity and mortality associated with these diseases. The rational use of antibiotics should also be practiced.

## Background

Diarrheal diseases are the major causes of morbidity and mortality in developing world [1]. The burden of diseases associated with intestinal parasitic infections and enteropathogenic bacteria is enormous [1-4]. Globally about two billion people are affected by intestinal parasites, of whom 300 million suffer from associated severe morbidity [2]. The high prevalence rates of the intestinal parasites are attributed largely to poor socio-economic status, poor sanitation, inadequate medical care and absence of safe and adequate water supplies [3]. Intestinal parasitic infections are among the major cause of diseases of public health problems in sub-Saharan Africa including Ethiopia [5]. Shigellosis is a highly infectious disease of world significance. Its prevalence is highest in tropical and subtropical parts of the world where living standards are very low and access to safe and adequate drinking water and proper excreta disposal systems are often limited [6]. *Salmonella *infections also remain as an important public health problem particularly in developing countries [7]. Like other developing nations, shigellosis and salmonellosis are among the common causes of morbidity and mortality in Ethiopia [6,8]. Moreover, emergence and spread of antibiotic resistance is posing serious problems in antimicrobial treatment worldwide [9].

*Escherichia coli O157:H7 *has emerged as an important food borne pathogen of considerable public health concern, because of the severity of infection which causes [10]. *E. coli *O157: H7 is one of the hundreds of strains of the bacterium enterohaemorrhagic *E. coli *and a pathogenic serotype. It has been documented that outbreak due to *E. coli O157: H7 *occurred in refugee camps in Mozambique, Swaziland and Malawi [11]. According to studies undertaken in United Kingdom, North America and elsewhere, *E. coli *O157:H7 is recognized as the major cause of haemorrhagic colitis and hemolytic uraemic syndrome [11-13]. Except a single study conducted from retail raw meat products which showed a 4.2% prevalence of *E. coli *O157:H7 among 738 meat specimens inspected [14], there has been no other report on the pathogen from human subjects in Ethiopia.

Understanding the magnitude of intestinal parasites, the prevalence and drug susceptibility pattern of enteropathogenic bacteria is important in designing public health intervention measures. Since studies addressing such issues are very scanty in northwest Ethiopia; the present study was aimed to assess the prevalence of intestinal parasites, *Shigella *and *Salmonella *species, and *E. coli *O157:H7 in patients who were presenting diarrhea at Gondar teaching hospital, northwest Ethiopia.

## Methods

A cross sectional study was conducted in Gondar teaching hospital, Gondar, Ethiopia between October 2006 and March 2007, and 384 consecutive patients presenting with diarrhea (passage of three or more loose stools per 24 hours) [15] were included during the study period. A structured questionnaire was utilized to collect socio-demographic characteristics and relevant clinical data of the patients. Patients who did take any antibiotics in the past four weeks were excluded.

Stool specimens were collected following the standard procedure [11]. Samples were then inoculated immediately on MacConkey and Salmonella-Shigella agar plates (Oxoid). The inoculated plates were incubated at 37°C aerobically for 24 hours. The plates were then examined for the presence or absence of visible bacterial colonies. The presence of non-lactose fermenting (NLF) colonies was taken as a presumptive diagnostic tool for *Shigella *and *Salmonella *species. The NLF colonies were further tested through a series of biochemical tests to identify *Shigella *and *Salmonella *species [11]. Antibiotic resistance testing of the *Shigella *and *Salmonella *species was conducted on Muller-Hinton agar (DIFCO) against the commonly used antibiotics: tetracycline (TTC, 30 μg), ampicillin (AMP, 30 μg), cotrimoxazole (SXT, 25 μg), gentamicin (GEN, 10 μg), chloramphenicol (CAF, 30 μg) and ciprofloxacin (CIP, 5 μg) following the single disc diffusion technique [16].

All stool samples were immediately cultured on Eosin Methylene Blue (EMB) agar (Oxoid) for primary screening of *E. coli *and incubated aerobically at 37°C for 24 hours. Suspected colonies of *E. coli*, a green metallic sheen on EMB, were further subcultured on Sorbitol MacConkey Agar (Oxoid) supplemented with 0.05 mg/liter cefixime and 2.5 mg/liter potassium tellurite (Oxoid) and incubated at 37°C for 24 hours. Following the incubation period, the agar plates were inspected for the presence of non-sorbitol fermenter colonies. All non-sorbitol fermenting colonies were further serotyped for *E. coli *O157:H7 with a commercial serologic kit following the manufacturer's instructions (Oxoid *E. coli *O157 Latex agglutination test, UK). The sensitivity and specificity of the kit is 100% and 99%, respectively. The latex beads were coated with antibodies, which bind to any O157:H7 antigens on the test organisms, forming a visible antigen- antibody precipitate [17]. Proper microbiological quality control was employed at each step of the procedure and American Type Culture Collection quality control strains of *Escherichia coli *ATCC 25922 and *Pseudomonas aeruginosa *ATCC 27853 were used for susceptibility testing.

Stool specimens were also processed and examined by direct microscopy for intestinal parasites. Modified acid-fast staining technique was employed to detect *Cryptosporidium parvum *and *Isospora belli *[18].

The data was entered and analyzed using SPSS version 13 packages. The relationships between proportion of intestinal parasitism and *Shigella *infections, and independent variables were analyzed using chi-square tests. *P*-value less than 0.05 was considered as statistically significant.

The study was reviewed and approved by the Institutional Ethical Review Board of the University of Gondar, Gondar, Ethiopia and informed consent was also obtained from the study subjects and/or guardians. Patients were treated as per the existing clinical practices of the health institution.

## Results

Three hundred eighty four diarrheal patients were included in this study. The mean ± SD age of the participants was 27.9 ± 18.3 years and 53.1% of study subjects were females. A quarter of the patients (25%) were children under 5 years. The over all prevalence of intestinal parasites in the present study was 36.5%. The predominant protozoan parasite detected was *Entamoeba histolytica/dispar *(7.3%) followed by *Giardia lamblia *(5.0%). Opportunistic protozoan parasites: C*ryptosporidium parvum *(1.8%) and *Isospora belli *(1.3%) were also detected. *Ascaris lumbricoides *was the dominant helminthic parasite identified (5.5%) followed by *Strongyloides stercoralis *(3.1%) and *Schistosoma mansoni *(3.1%) (Table 1). Multiple infections with two and three intestinal parasites was detected in 3.6% (Table 2) and 2.6% (Table 3), respectively.

**Table 1:** Intestinal parasites in diarrheal patients at Gondar teaching hospital, Gondar, Ethiopia, October 2006 to March 2007.

Intestinal parasites	Male (n = 180) No. (%)	Female(n = 204) No. (%)	Total (n = 384) No. (%)	*P *value
*Entamoeba histolytica/dispar*	8(4.4)	20(9.8)	28(7.3)	0.044
*Giardia lamblia*	5 (2.8)	14(6.9)	19(5.0)	0.066
*Ascaris lumbricoides*	10(5.6)	11 (5.4)	21(5.5)	1.00
*Strongyloides stercoralis*	6 (3.3)	6 (2.9)	12(3.1)	0.82
*Schistosoma mansoni*	6 (3.3)	6(2.9)	12(3.1)	0.82
Hookworm infection	3 (1.7)	4(2.0)	7(1.8)	1.00^♣^
*Hymenolepis *species	3 (1.7)	2 (1.0)	5(1.3)	0.67^♣^
*Cryptosporidium parvum*	4 (2.2)	3(1.5)	7(1.8)	0.71^♣^
*Isospora belli*	0 (0)	5(2.5)	5(1.3)	0.063^♣^
Multiple infections	12 (6.7)	12 (5.9)	24 (6.3)	0.751
Overall prevalence	57 (31.7)	83(40.8)	140 (36.5)	0.066

^♣^P value from Fisher's exact test

**Table 2:** Patients harboring double infections and types of parasite combinations in diarrheal patients at Gondar teaching hospital, October 2006 to March 2007.

Parasite combinations	Male (n = 180)	Female (n = 204)	Total (n = 384)
	No. (%)	No. (%)	No. (%)
Al, Sst	0 (0)	1 (0.49)	1 (0.26)
Al, Sm	0 (0)	1 (0.49)	1 (0.26)
Al, Eh,	0 (0)	1 (0.49)	1 (0.26)
Al, Ib	1 (0.55)	0 (0)	1(0.26)
Al, Gl	1 (0.55)	1 (0.49)	2 (0.52)
Eh, Gl	1 (0.55)	0 (0)	1 (0.26)
Eh, Sm	2 (1.1)	0 (0)	2 (0.52)
Eh, Sst	0 (0)	2 (0.99)	2 (0.52)
Sm, Gl	1 (0.55)	1 (0.49)	2 (0.52)
Gl, Ib	1 (0.55)	0 (0)	1 (0.26)
Total	7(3.9)	7(3.4)	14(3.6)

Keys: Al- *Ascaris lumbricoides*, Sst- *Strongyloides stercoralis*, Hy spp.-*Hymenolepis *species, Sm- *Schistosoma mansoni*, Eh- *Entamoeba histolytica*, Gl- *Giardia lamblia*, Cp- *Cryptosporidium parvum*, Ib-*Isospora belli*, Hw-Hookworm.

**Table 3:** Frequency of triple infections and types of parasite combinations among diarrheal patients at Gondar teaching hospital, Gondar, Ethiopia, October 2006 to March 2007.

Parasite combinations	Male (n = 180)	Female (n = 204)	Total (n = 384)
	No. (%)	No. (%)	No. (%)
Al, Sst, Gl	0 (0)	1 (0.49)	1 (0.26)
Al, Eh, Sm	0 (0)	1 (0.49)	1 (0.26)
Al, Sst, Hw	1 (0.56)	0 (0)	1 (0.26)
Al, Eh, Hw	1 (0.56)	0 (0)	1 (0.26)
Al, Gl, Cp	0 (0)	1 (0.49)	1 (0.26)
Al, Hy spp., Cp	1 (0.56)	0 (0)	1 (0.26)
Sst, Gl, Hw	2 (1.1)	0 (0)	2 (0.52)
Eh, Gl, Hw	0 (0)	2 (0.98)	2 (0.52)
Total	5 (2.8)	5 (2.5)	10 (2.6)

Keys: As indicated in table 2

The prevalence of intestinal parasite was significantly higher (*P *= 0.013) in patients who live in rural (41.9%) than urban area (29.6%), in patients who were using well and spring water (62.9%) than who were using pipe (30.7%) for household consumption (*P <*0.0001). There was a statistically significant relationship between presence of intestinal parasites and hand washing practice after visiting toilet either irregularly with soap and without soap or not at all (69.1%) than patients who were washing their hands regularly with soap (28.7%), (*P *= 0.005). Statistically significant difference was also observed between presence of intestinal parasites and nausea (42.9%) than patients who did not have nausea (28.5%), (*P *= 0.003).

Statistically significant associations were also observed between infections with *Shigella *species and patients who were using well and spring water (37.6%) than pipe water (8.5%) for household consumption,(*P *= 0.004), and in patients who had dysentery and mucoid stool (66.4%) than patients who had watery stool(7.9%), (*P <*0.0001)(Table 4). No statistically significant difference was observed between occurrence of intestinal parasites, and availability of toilet, level of education, fever and appearance of the stool. Similarly there was no significant association between *Shigella *infections and variables such as residence, methods of hand washing, level of education, availability of toilet, nausea, and fever (*P >*0.05),(Table 4).

**Table 4:** Socio-demographic and clinical characteristics of diarrheal patients at Gondar teaching hospital, Gondar, Ethiopia, October 2006 to March 2007.

Variables	Parasite positive No (%)	Parasite negative No. (%)	*P*-value	Shigella spp. positive No (%)	Shigella spp. negative No. (%)	*P*-value
Residence						
Urban	50 (29.6)	119 (70.4)	0.013	25 (14.8)	144 (85.2)	0.70
Rural	90 (41.9)	125 (58.1)		35 (16.3)	180 (83.7)	
Source of water						
Pipe	47 (30.7)	106 (69.3)	<0.0001	13 (8.5)	140 (91.5)	0.004
Well	85 (48.9)	89 (51.1)		38 (21.8)	136 (78.2)	
Spring	8 (14.0)	49 (86.0)		9 (15.8)	48 (84.2)	
Availability of toilet						
Yes	66 (32.8)	135 (67.2)	0.122	33 (16.4)	168 (83.6)	0.70
No	74 (40.4)	109 (59.6)		27 (14.8))	156 (85.2)	
Hand washing after latrine						
Regularly with soap	43 (28.7)	107 (71.3)	0.005	24 (16.0)	126 (84.0)	0.90
Irregularly with soap	89 (44.1)	113 (55.9)		32 (15.8)	170 (84.2)	
Without soap/not at all	8 (25.0)	24 (75.0)		4 (12.5)	28 (87.5)	
Level of education						
Illiterate	52 (32.9)	106 (67.1)	0.70	24 (15.2)	134(84.8)	0.51
Primary school	54(38.8)	85 (61.2)		20(14.4)	119(85.6)	
Secondary school	32 (39.5)	49 (60.5)		16(19.8)	65(80.2)	
Others	2 (33.3)	4 (66.7)		0(0)	6(100)	
Appearance of stool						
Watery	105 (37.5)	175 (62.5)	0.70	22 (7.9)	258 (92.1)	< 0.0001
Dysentery	24 (34.8)	45 (65.2)		30 (43.5)	39 (56.5)	
Mucoid	11 (31.4)	24 (68.6)		8 (22.9)	27 (77.1)	
Nausea						
Yes	91 (42.9)	121 (57.1)	0.003	28 (13.2)	184(86.8)	0.20
No	49 (28.5)	123 (71.5)		32 (18.6)	140(81.4)	
Fever						
Yes	28 (39.4)	43 (60.6)	0.60	15 (21.1)	56 (78.9)	0.20
No	112 (35.8)	201 (64.2)		45 (14.4)	268 (85.6)	

*Shigella *species were isolated from 15.6% of the stool samples. Among patients who had *Shigella *infections, 18.3% were co-infected with intestinal parasites. The dominant parasite detected in these co- infected patients was *I. belli *(20%) followed by *A. lumbricoides *(19%), *G. lamblia *(15.8%) and *E. histolytica/dispar *(14.3%). Resistance to TTC, AMP, SXT, CAF, GEN, and CIP was observed in 85, 80, 76.7, 48.3, 10, and 8.3%, of the *Shigella *isolates, respectively. Forty, 33.3, 3.3 and 3.3% of the *Shigella *isolates were found to be resistant to 3, 4, 5 and 6 commonly used antibiotics, respectively (Table 5).

**Table 5:** Multiple drug resistance patterns of *Shigella *and *Salmonella *species isolated from diarrheal patients at Gondar teaching hospital, Gondar, Ethiopia, October 2006 to March 2007.

Types of resistance	Resistant isolates No. (%)
*Shigella *species Resistance to 3 antibiotics	
AM-SXT-TTC	21 (35)
CAF-SXT-TTC	2 (3.3)
AMP-CAF-TTC	1 (1.7)
Resistance to 4 antibiotics	
AMP-CAF-SXT-TTC	19 (31.7)
AM-SXT-TTC-CIP	1 (1.7)
Resistance to 5 antibiotics	
AMP-CIP-GEN-SXT-TTC	2 (3.3)
Resistance to 6 antibiotics	
AMP-GEN-SXT-TTC-CAF-CIP	2 (3.3)
*Salmonella *species	
Resistance to 3 antibiotics	
AM-SXT-TTC	2(33.3)
Resistance to 4 antibiotics	
AM-SXT-CAF-TTC	1(16.7)
SXT-CAF-TTC-GEN	1(16.7)

*: AMP: ampicillin; GEN: gentamicin; SXT: cotrimoxazole; CAF: chloramphenicol; TTC:tetracycline; CIP: ciprofloxacin

*Salmonella *species were isolated from six diarrheic (1.6%) patients. Of the *Salmonella *isolates, 83.3% (5/6) were resistant for AMP and TTC. Sixty seven, 50, and 16.7% of the isolates were resistant to SXT, CAF and GEN, respectively. The majority *Salmonella *isolates were resistance for 3 or 4 commonly used antibiotics (Table 5). All of the *Salmonella *isolates were sensitive to CIP. No *E. coli O157: H7 *was detected from stool samples of all (0%) the diarrheic patients.

## Discussion

In this cross-sectional study among diarrheal patients in Gondar teaching hospital, northwest Ethiopia, the overall prevalence of intestinal parasites in stool samples was found to be 36.5%. This finding was consistent with previous study conducted in southwest Ethiopia [19] and with a report from Yemen [20]. However, our finding was lower compared to the studies undertaken in central Ethiopia and South Africa [21,22]. These could be due to the differences in hygiene practices of the populations, environmental and host factors. The methods used for detection of the parasites could also attribute to the observed difference.

*E. histolytica/dispar *was the predominant protozoan parasite (7.3%) isolated from stool of the diarrheic subjects. This report was comparable to the study conducted by Al-Mohammed et al [23]. The occurrences of A. *lumbricoides *(5.5%), *G. lamblia *(5%) and *S. stercoralis *(3.1%) detected in the current study were in agreement with a study conducted in southwest Ethiopia [19]. The rate of protozoan opportunistic infections: *C. parvum *(1.8%) *and I. belli *(1.3%) in the present study were low compared with previous study done in central Ethiopia [21]. This discrepancy could be due to the methods used to detect the parasites and/or low rate of those parasites in the study area. However, similar rate of *C. parvum *was reported in a study done by Lee et al [24].

The rate of *S. mansoni *(3.1%) and hookworm infection (1.8%) observed in the study are in line with reports done elsewhere [25,26]. Similarly, the 1.6% of *Hymenolepis *species diagnosed in the study was also in accordance with a study conducted in Yemen [20]. Multiple infections with intestinal parasites occurred in 6.3% of patients and this rate was comparable with a report from Nigeria [27].

Our result revealed that significantly higher parasitic infections were observed in patients who live in rural than those who live in urban area. This difference may occur due to lack of awareness towards general hygiene practices in rural compared to patients who live in urban area. A similar result was also found in a study undertaken in Yemen [20]. With respect to water sources for household consumption, patients who were using well and spring water for daily household consumption had higher rate of intestinal parasites and *Shigella *species than patients who were using pipe water. This variation may be due to the fact that those water sources were not protected, which pose significant health problems to acquire the infections. Patients who used to wash their hands after visiting toilet either irregularly with soap and without soap or not at all had significant higher intestinal parasites and this finding was consistent with a study conducted in Uganda [28].

In the study, *Shigella *species were isolated from 15.6% of the diarrheal patients. This result was consistent with studies done in Kenya and Tanzania where 16% and 14% *Shigella *isolates have been reported, respectively [29,30]. However, our finding was lower compared to a 34.6% prevalence of *Shigella *species isolated from a study done in Awassa, southern Ethiopia [31]. The difference might be due to the nature of the public water supply scheme in the setting which is from Lake Awassa and supposed to be more contaminated than the public water supply system of Gondar town which is a protected surface water system. Similarly, the 1.6% isolation rate of *Salmonella *species in our report was comparable with previous reports in northwest and northern Ethiopia in which 1% and 2.01% of *Salmonella *isolates reported, respectively [32,33].

Antimicrobial resistance to one or more antibiotics was very high among the *Shigella *species isolated in the study (88%). Multiple resistances (resistance for two up to six commonly used antibiotics) were observed in 80% of the *Shigella *species isolated. This finding was in line with a study conducted in southern Ethiopia where 82% isolates were found to be multi drug resistant [31]. Other studies from Ethiopia also showed increased antibiotic resistance among *Shigella *isolates [32,34,35]. In the current study, *Shigella *isolates were resistant to TTC (85%), AMP (80%), SXT (76.7%) and CAF (48.3%) and these findings were comparable with previous studies conducted in Ethiopia [31,34,35] and other African countries [36,37]. Ten percent of the Shigella isolates were resistant to GEN and this result was in agreement with a study conducted in Nigeria [36]. Comparatively high rate of resistance to CIP (8.3%) was observed in the present study as compared to previous report in which 3.1% of *Shigella *isolates were resistant to CIP [38]. This high resistance rate might reflect the indiscriminate and widespread uses of the antibiotics in public health practices since the society in the setting have easy access to different antibiotics and could buy the antibiotics without prescription [39]. However, 16% and 28.3% of *Shigella *isolates resistance to CIP were reported in South Africa and Nepal, respectively [22,40]. The patterns of resistance for the isolated *Salmonella *species in this study were consistent with previous studies conducted in South Africa, Ethiopia and Mexico [22,34,41]. The absence of *Salmonella *isolates resistance for CIP in the present study suggests that CIP could be used as a drug of choose for treating *Salmonella *infections in the absence of drug susceptibility test.

The absence of *E. coli *O157:H7 in our study subjects was comparable with study conducted in Uganda [42]. This absence might be due to the feeding habit of the study population. *E. coli *O157:H7 strains were first detected following the ingestion of hamburgers in the United States in 1982 [43] and out breaks were occurred in United States relating in acidic foods such as mayonnaise and apple cider have underscored the unusual acid tolerance of this organism [44,45]. It is worthy to note that, many of the outbreaks that had occurred around the world were more or less related with fast foods like hamburger and acid foods such as apple-cider and mayonnaise, which are not commonly consumed by our study population and inaccessible of these foods to the study subjects. Absence of *E. coli *O157:H7 also reported from studies conducted in Spain and Italy [46,47]. On the contrary a single case and 5.4% of *E. coli O157:H7 *identified from reports done in South Africa and Nigeria, respectively [48,49].

## Conclusions

Diarrheal patients included in this study had high prevalence of intestinal parasites and *Shigella *species, low prevalence of *Salmonella *species and no *E. coli *0157:H7. The *Shigella *and *Salmonella *species showed very high level of antimicrobial resistance. Interventions including health education on personal hygiene, provision of safe and adequate water supply to the community and in depth studies of possible epidemiologic associations among diarrhea, intestinal parasitosis and bacterial infections in the region are imperative and the rational use of antibiotics should also be practiced. The absence of *E. coli *O157:H7 might show limited circulation or absence of this strain in the area and may imply screening of diarrheic stools for pathogenic *E. coli *O157:H7 in routine clinical practice in the area might not be necessary. However, in-depth multi-centric studies are required to substantiate the present finding.

Nevertheless, the study has the following limitations: we did not speciate *Shigella *and *Salmonella *isolates. The speciation of the isolates would have been more valuable if the biotyping of *Shigella *and *Salmonella* was done, however, still the finding is important in the setting, Second the status of each patient was not known for HIV infection, this would be useful to correlate the results to HIV infected and non-infected subjects. Moreover, based on our objectives we only intended to investigate intestinal parasites, *Shigella *and *Salmonella *species and *E.coli*O157:H7 as causative agents for diarrhoea as a result all causes of diarrhea among the patients were not studied.

## Competing interests

The authors declare that they have no competing interests.

## Authors' contributions

KH, Principal investigator of the study, study design, data collection, laboratory work, and data analysis; AK, Study design and data analysis; AM and FM, study design and laboratory work; FB, NW, TF, data collection and laboratory work; SG, YB, AG, BA, SY, YW, MT, data collection, laboratory work and supervision of the work; DR, AB, AMU, supervision of the work; all authors contributed to the write up. All authors read and approved the final manuscript.
